# Primary Petit Hernia: From Diagnosis to Open Surgical Approach

**DOI:** 10.7759/cureus.35789

**Published:** 2023-03-05

**Authors:** Duarte Gil Alves, Jessica Sousa, Catarina Rodrigues, Sara Silva, Rómulo Ribeiro

**Affiliations:** 1 General Surgery, Hospital Dr. Nélio Mendonça, Funchal, PRT; 2 Radiology, Centro Hospitalar Universitario do Porto EPE, Porto, PRT

**Keywords:** lumbar hernia, petit's hernia, surgical mesh, open hernia surgery, abdominal hernia, primary lumbar hernia

## Abstract

Lumbar hernias are quite rare, even more so when primary or of spontaneous nature. These defects in the lumbar region demand a comprehensive knowledge of the anatomy of the lateral abdominal wall and paraspinal muscles. Given the proximity of bone structures, they can pose a surgical challenge when trying to achieve an ideal dissection and appropriate mesh overlap. The authors report the case of a primary Petit’s hernia that underwent an open anterior surgical approach with the use of a preperitoneal mesh. In addition to the described surgical technique, the article also aims to detail the diagnosis and anatomic classification of this rare pathology.

## Introduction

Lumbar hernias are defects that occur through the lumbar region bordered by the 12th rib superiorly, the iliac crest inferiorly, the paraspinal muscles medially, and the external oblique muscle laterally [1]. According to their etiopathogenesis, they can be classified as congenital or acquired. Acquired lumbar hernias can be further divided into primary or secondary to a triggering event such as trauma, previous surgeries, or infection [2].

Primary lumbar hernias can occur through the superior lumbar triangle (Grynfeltt-Lesshaf) bordered by the 12th rib, the quadratus lumborum, and the interior oblique muscle. On the other hand, if they occur through the inferior lumbar triangle bordered by the iliac crest, the latissimus dorsi, and the external oblique muscle, then they should be referred to as Petit’s hernia [3].

With only a few hundred cases reported, lumbar hernias are extremely rare defects [4]. Therefore, neither international consensus nor randomized controlled trials exist regarding the appropriate surgical approach.

## Case presentation

An 83-year-old female patient, with a past medical history of hypertension and atrial fibrillation, presented to the emergency department. The chief complaint was constant and non-radiating pain in the right lumbar area for the past two days associated with a bulge. The patient denied any trauma, previous surgery, or changes in bowel movements.

On physical examination, the patient was found to have a palpable and reducible mass on the right lateral abdominal wall. An ultrasound was performed for further evaluation. It revealed the presence of a right lumbar hernia with a sac containing adipose tissue and ascitic fluid. Neither signs of incarceration nor dilated intestinal loops were identified. The patient was discharged and elective surgery was scheduled.

**Figure 1:**
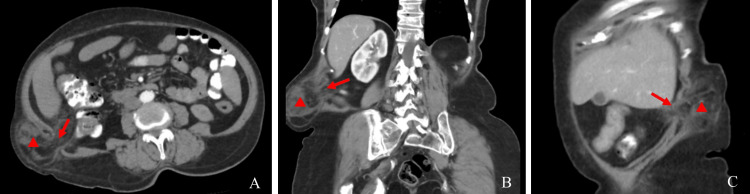
Axial (A), coronal (B), and sagittal (C) enhanced CT acquisitions showing a right inferior lumbar hernia with a neck size of 3.5 cm (arrow) and a hernia sac measuring 8.1cm by 4.9 cm containing adipose tissue and small bowel loops (triangle).

In order to better characterize the size and location of the hernia, thus allowing to formulate a proper surgical plan beforehand, a computed tomography (CT) of the abdomen and pelvis was performed. It showed a right inferior lumbar hernia with a neck size of 3.5 cm and a hernia sac measuring 8.1 cm by 4.9 cm (Figure 1).

**Figure 2:**
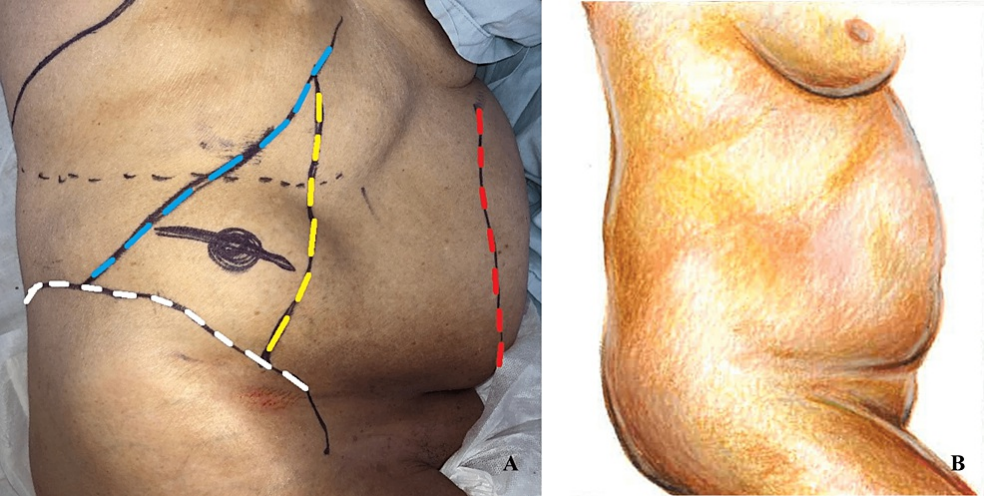
(A) Lateral left decubitus position showing the Petit’s triangle (inferior lumbar triangle) limited inferiorly by the iliac crest (white dotted line), anteriorly by the posterior border of the external oblique muscle (yellow dotted line) and posteriorly by the lateral border of the latissimus dorsi muscle (blue dotted line). The floor is composed by the internal oblique muscle. The lateral border of the rectus abdominis muscle is marked by the red dotted line; (B) An illustration of the patient’s position.

**Figure 3:**
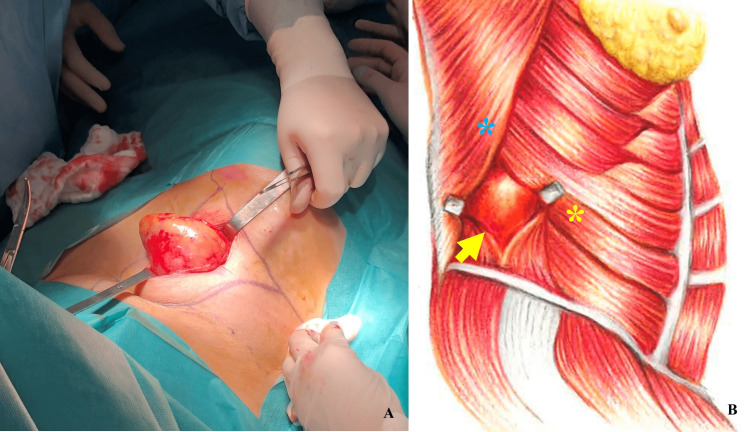
(A) Hernia sac freed from the surrounding fascial edges; (B) An illustration showing the hernia sac (yellow arrow) protruding superior to the iliac crest, posterior to the external oblique muscle (yellow asterisk) and anterior to the latissimus dorsi muscle (blue asterisk).

After an explanation of the planned procedure, the patient gave consent and was put under general anaesthesia. She was then placed in a left lateral decubitus position (Figure 2). A surgical incision was made over the defect and dissection proceeded around the hernia sac, freeing it from the surrounding fascia (Figure 3).

**Figure 4:**
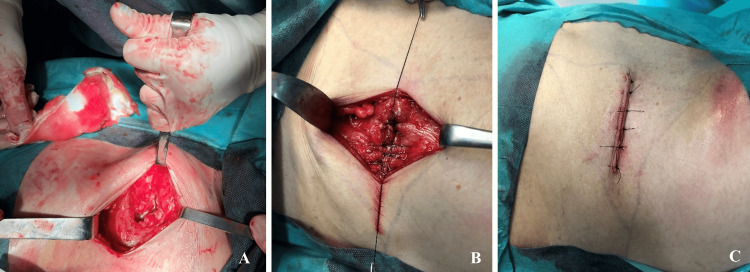
(A) Hernia reduced; (B) Primary closure of the muscle plane restoring the abdominal wall anatomy; (C) Final skin closure.

Once all the adhesions had been removed, the surgeons were able to reduce the hernia contents while keeping the integrity of the hernia sac. Only then, did they manage to dissect the preperitoneal space, allowing for a mesh overlap of at least 5 cm in all directions. A double-layer polypropylene macroporous mesh, 10 cm by 10 cm, was placed in the preperitoneal plane and anchored with slowly absorbable sutures. Even though the peritoneum was inspected for any defects, the surgeons chose to use a double-layer mesh as the peritoneum was thin at this level and they wanted to ensure there was a low risk of bowel adhesions. The muscle plane was then closed by reapproximating the abdominal wall under physiologic tension, restoring the normal anatomy and its function (Figure 4). The patient evolved favourably and was discharged the following day. After four years of follow-up, the patient remains asymptomatic.

## Discussion

The most common clinical presentation of a lumbar hernia is pain associated with bulging [5]. Although uncommon, the possibility of bowel obstruction or incarceration must be ruled out [6].

CT scan of the abdomen and pelvis should be preoperatively performed. Not only is it a great imaging modality to confirm the diagnosis, it also allows the assessment of the anatomical relationships and surgical technique planning [7]. Alternatively, in selected patients, magnetic resonance imaging (MRI) can be performed and is able to successfully characterize the lumbar hernia’s anatomy [8].

When symptomatic, lumbar hernias should be surgically repaired [9]. The use of mesh is required, either by an open anterior preperitoneal approach or by a laparoscopic posterior intraperitoneal or preperitoneal approach [10]. Since minimally invasive surgery is traditionally associated with less postoperative pain, reduced hospital stay, and fewer complications, smaller defects should be repaired by laparoscopy or robotic surgery when surgeon’s experience and resources allow for it [11]. Although a robotic approach to lumbar hernias seems feasible and beneficial, the rarity of this entity and the scarcity of data limits the recommendation of a preferred repair method [12,13]. Prior to mesh placement, dissection should be performed in all directions to allow for a mesh overlap of at least 5 cm [14]. Considering the close proximity to bone structures and the complexity of the surgical repair, it may be advisable to forward patients to experienced abdominal wall centres [10].

## Conclusions

Despite lumbar hernias being so rare, all surgeons must have an in-depth understanding of their management approach, as they may present in an acute setting requiring an emergent repair. If possible, a preoperative CT is recommended since it can assist in the formulation of a surgical approach.

A tailored approach should be chosen, according to the lumbar hernia’s features and the surgeon’s expertise. Patient referral to specialized abdominal wall centres should be considered.
